# Dual-Emission Fluorescent Microspheres for the Detection of Biothiols and Hg^2+^

**DOI:** 10.3390/ma11112232

**Published:** 2018-11-09

**Authors:** Jiahui Wang, Hao Zhang, Ruifang Guan

**Affiliations:** School of Material Science and Engineering, University of Jinan, Jinan 250000, China; 17862917099@163.com (J.W.); 15069079873@163.com (H.Z.)

**Keywords:** dual-emission, silica microspheres, fluorescence

## Abstract

Dual-emission nanosensor for Hg^2+^ detection was prepared by coupling CA-AEAPMS on the surface of RBS-doped modified silica microspheres. The CA-AEAPMS was synthesized by using *N*-(β-aminoethyl)-γ-aminopropyl methyldimethoxysilane (AEAPMS) and citric acid as the main raw material. The obtained nanosensor showed characteristic fluorescence emissions of Rhodamine B (red) and CA-AEAPMS (blue) under a single excitation wavelength (360 nm). Upon binding to Hg^2+^, only the fluorescence of CA-AEAPMS was quenched, resulting in the ratiometric fluorescence response of the dual-emission silica microspheres. This ratiometric nanosensor exhibited good selectivity to Hg^2+^ over other metal ions, because of the amide groups on the surface of CA-AEAPMS serving as the Hg^2+^ recognition sites. The ratio of F_450_/F_580_ linearly decreased with the increasing of Hg^2+^ concentration in the range of 0 to 3 × 10^−6^ M, and a detection limit was as low as 97 nM was achieved. Then, the addition of three thiol-containing amino acids (Cys, Hcy, GSH) to the quenched fluorescence solution with Hg^2+^ can restore the fluorescence, and the detection limits of the three biothiols (Cys, Hcy, GSH) are 0.133 μM, 0.086 μM, and 0.123 μM, respectively.

## 1. Introduction

Mercury is a kind of cumulative harmful metal element known for its excessive toxicity it derives from its high affinity for thiol groups in enzymes and proteins, leading to cell dysfunction and subsequent physical illness. It usually enters the body through the atmosphere and water. Excessive mercury often cause poisoning or various incurable diseases [1,2,3,4,5].

Cysteine (Cys), homocysteine (Hcy), reduced glutathione (GSH), and other biothiols, as the most small bio-thiol molecules in the cell, play important roles in human physiological activities [6,7,8], including intracellular redox reactions, foreign body metabolism, signal transduction, and gene regulation, etc. Reduced glutathione plays a key role in cell growth and redox balance maintaining normal cell function. Abnormal cystine concentrations often lead to diseases such as cancer, Alzheimer’s disease, and cardiovascular disease [9,10,11].

Detection and quantification of mercury ions and biothiols such as Cys, Hcy, GSH in biological and environmental samples contribute to monitoring and preventing disease [9,10,11]. Therefore, it is highly desirable to develop relatively inexpensive and reliable methods to detect them. On the other hand, ratiometric fluorescent probes have important applications in ion detection because of their high sensitivity, good selectivity, convenient synthesis, low cost, and wide application range [12,13,14]. Ratiometric fluorescent probes often comprise two kinds of fluorescent substances at different emission wavelengths, thus eliminating the influence of the external environment on the test results, making the results more reliable [15,16,17,18,19,20].

In this paper, dual-emission fluorescent microspheres were prepared using amide-based fluorescent small molecules (CA-AEAPMS) synthesized in our laboratory [21] and RBS (RB-APTES) [22]. A variety of ions were detected using prepared dual-emission fluorescent SiO_2_ microspheres, and it was found that they can applied to detect Hg^2+^ and Cys, Hcy, and GSH quickly and sensitively. The detection of Hg^2+^ is based on its quenching effect on small fluorescent molecules, while the detection of biothiol is due to the interaction of biothiol with Hg^2+^ over the small fluorescent molecules and the removal of Hg^2+^ from the surface of the fluorescent microspheres [5], so that the fluorescence of the system can be recovered.

## 2. Materials and Methods

### 2.1. Reagents and Materials

*N*-(β-aminoethyl)-γ-aminopropyl methyl-dimethoxysilane (AEAPMS), anhydrous citric acid (CA), aminopropyltriethoxysilane (APTES), thionyl chloride rhodamine B, tetrahydrofuran, and TEOS and other reagents were purchased from Aladdin (Shanghai, China). Ethanol was purchased from Daliang Town Industrial Park (Tianjin, China). Ammonia was purchased from Kermel (Tianjin, China). Deionized water was used throughout all the experiments.

Fluorescence measurements were performed on a Fluoromax-4 fluorescence spectrometer. Low temperature reaction was recorded in a low-temperature reaction bath DHJF-4005, ultrasonic dispersion in an ultrasound machine KQ3200DB, and centrifugation in a TG16G high-speed centrifuge.

### 2.2. Preparation of RBS-Doped Silica Microspheres

First, RB-APTES (RBS) was synthesized according to the procedure reported elsewhere [22]. A total of 0.48 g of RB was dissolved in 15 mL of chloroform with stirring the solution and heating to the boiling point of chloroform (61 °C). Next, 0.233 mL of APTES was added to react for 30 min. The obtained product was heated to 70 °C and rotary evaporation crystallization was needed to obtain RBS (RB-APTES) for the next use. Next, 50 mL of absolute ethanol, 2.5 mL of deionized water, and 1.5 mL of ammonia was added to the beaker and stirred for 30 min at room temperature to mix evenly. 1 mL of TEOS (ethyl silicate) and 0.05 g of RBS were slowly added dropwise to the above solution, and the polyethylene film was sealed after the dropwise addition. The mixture was stirred for 6 h, and then was purified by centrifugal washing and drying to obtain RBS-doped fluorescent SiO_2_ microspheres.

### 2.3. Preparation of CA-AEAPMS Coated Dual-Emission Silica Microspheres

Next, 0.5 g of anhydrous citric acid was dissolved in 20 mL of tetrahydrofuran, then 0.7 mL of dichlorosulfoxide was added drop-by-drop and the mixture was stirred for 12 h at −5 °C. The resulting product was washed three times with azeotropic purification of hexane and tetrahydrofuran to remove excess dichlorosulfoxide to obtain CA-CL (citric acid chloride). Then, 0.1 g of RBS-doped SiO_2_ microspheres was dissolved in 50 mL of absolute ethanol. After 5 mL of deionized water, 2 mL of ammonia were added, and the solution was stirred at room temperature for 10 min, then 0.5 mL TEOS was added. After stirring for 30 min, 0.05 mL of AEAPMS was added and stirred for 12 h at room temperature, then centrifuged, washed, and dried to obtain surface-modified fluorescent SiO_2_ microspheres. The obtained microspheres were ultrasonically dispersed in 50 mL of tetrahydrofuran for 1 h, then added an appropriate amount of CA-CL. The CA-CL reacted with the AEAPMS attached to the surface of the microspheres to form CA-AEAPMS [21]. The mixture was stirred for 6 h, and then was purified by centrifugal washing and drying. The dried fluorescent microspheres were placed in an oven and heated at 115 °C for 2 h to obtain dual-emission fluorescent SiO_2_ microspheres with overall blue fluorescence.

### 2.4. Fluorescence Assay of Hg^2+^

The fluorescence detection was performed in deionized water. Typically, 1.25 mg of dual-emission silica microspheres was redispersed into 5.0 mL of deionized water, which was used as the working solution of ratiometric nanosensor. Hg^2+^ was added directly to the nanosensor solution and detected on a Fluoromax-4 fluorescence spectrometer. The excitation wavelength for fluorescence measurements was 360 nm.

### 2.5. Selectivity and Interference Testing

The stock solutions of perchlorate salts (FeCl_3_·6H_2_O, HgCl_2_, CuCl_2_·2H_2_O, CoCl_3_·6H_2_O, AlCl_3_, CaCl_2_, NiCl_2_·6H_2_O, ZnCl_2_, NaCl, KCl, MgCl_2_, BaCl_2_, AgCl) were prepared in ultrapure water at the concentration of 10.0 mM. Stock solutions (10 mM) of various anions ions (Br^−^, I^−^, CO_3_^2−^,) were prepared by dissolving their sodium salts in deionized water.

## 3. Results and Discussion

### 3.1. Preparation and Characterization of the Dual-Emission Silica Microspheres

**Scheme 1:**
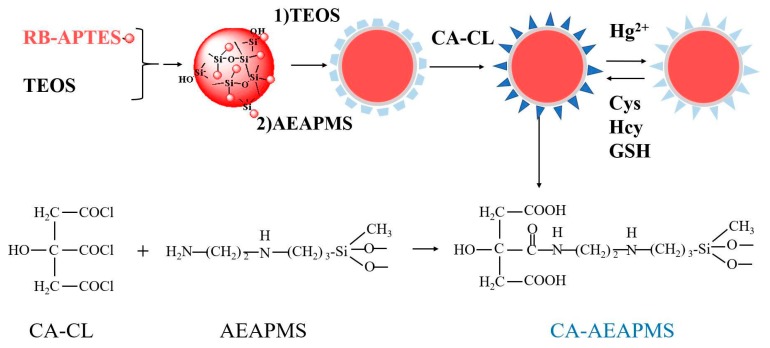
Schematic illustration of the preparation of dual-emission silica microspheres and reaction with Hg^2+^ and biothiols.

The preparation and sensing mechanism of the ratiometric nanosensor are shown in Scheme 1. First, RBS-doped silica microspheres (red) were prepared by using RBS and TEOS by a Stöber method. A thin layer of silica was then coated over the red silica core. There is a spectral overlap between the emission of CA-AEAPMS and the absorption of RBS, and the presence of a silicon shell can effectively isolate the two phosphors from fluorescence resonance energy transfer (FRET) as much as possible. Lastly, CA-CL was reacted with the amino group on the surface of the RBS-doped silica microspheres to form CA-AEAPMS (citric amide). The obtained silica microspheres showed two emission peaks at 450 nm and 580 nm due to the CA-AEAPMS emission and RBS emission.

**Figure 1:**
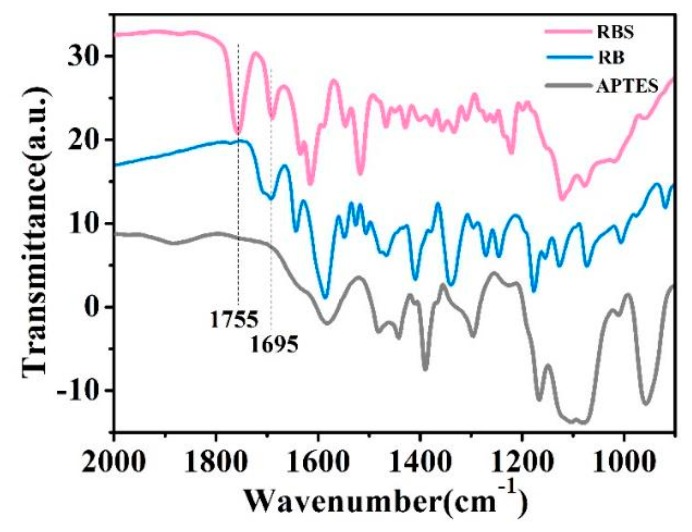
FTIR spectra of RBS (RB-APTES), RB (rhodamine B) and APTES (aminopropyltriethoxysilane).

**Figure 2:**
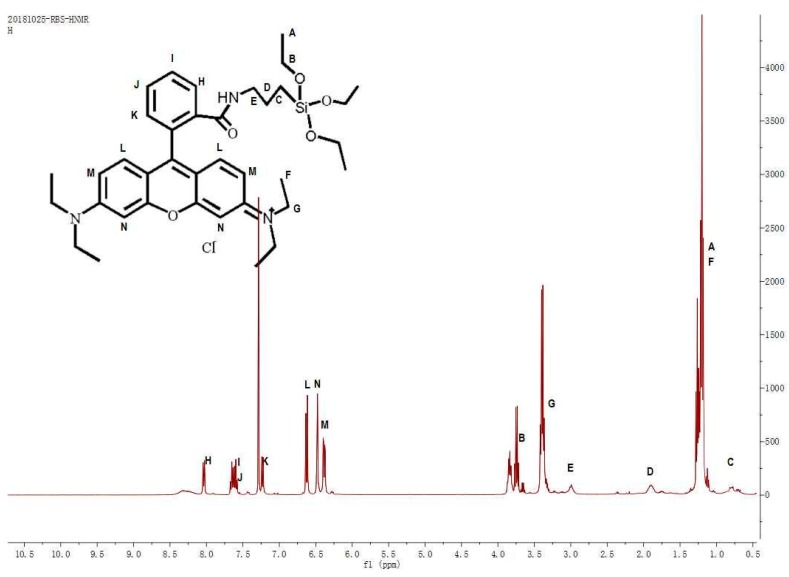
^1^H NMR spectrum of RBS in CDCl_3_.

Firstly, the organic dye RBS was structurally characterized. It can be seen from the comparison of the FTIR spectrum in Figure 1 that the carbonyl stretching vibration peak of RB at 1695 cm^−1^ was converted to 1755 cm^−1^ after condensation with APTES, which indicates a change of the COOH group of RB to CONH in the product. The ^1^H NMR spectrum of RBS shown in Figure 2 also illustrates the successful synthesis of RBS.

**Figure 3:**
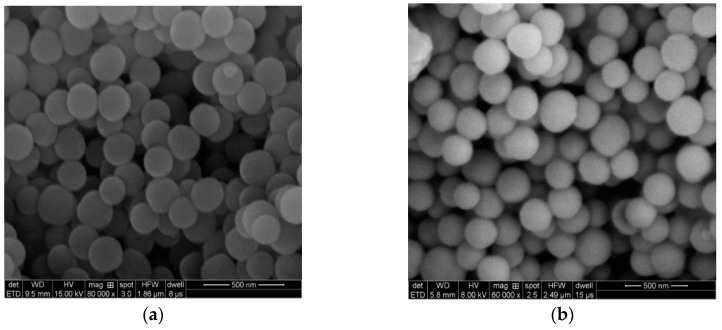
SEM images of the (**a**) RBS-doped silica microspheres, and (**b**) dual-emission silica microspheres.

The SEM results showed that the average diameter of the RBS-doped silica microspheres was about 160 nm (Figure 3a). After the silica microspheres were coated with CA-AEAPMS, the average diameter of the silica microspheres increased to about 200 nm (Figure 3b), suggesting the successful coating of CA-AEAPMS on the surface of RBS-doped silica microspheres.

**Figure 4:**
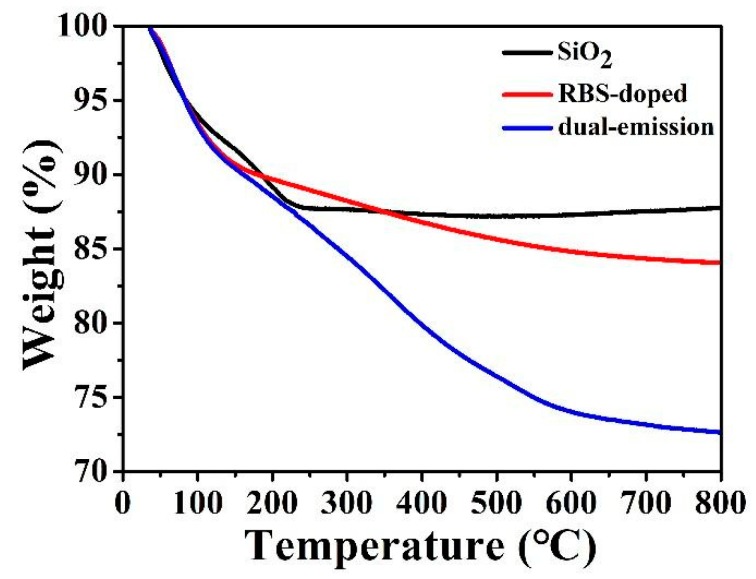
Thermogravimetric analysis (TGA) result of SiO_2_ microspheres, RBS-doped silica microspheres, and dual-emission fluorescent microspheres.

Thermogravimetric analysis (TGA) in Figure 4 showed that the total weight loss of SiO_2_ microspheres was about 12% at 35–230 °C; the total weight loss of RBS-doped silica microspheres at 35–800 °C was about 16%; the total weight loss of dual-emission fluorescent microspheres at 35–800 °C was about 28%. The weight loss during heating is basically divided into two stages. The weight loss before 100 °C is mainly the evaporation of water molecules and residual solvents adsorbed on the surface of the microspheres, and the weight loss is less, about 7%. After 100 °C, it is mainly decomposition of doped RBS. The additional 4% weight loss detected by the red fluorescent microspheres can be attributed to the thermal degradation of the organic fluorescent dye RBS in the silica matrix, and the additional 12% weight loss of dual-emission fluorescent microspheres can be attributed to thermal degradation of CA-AEAPMS on the surface of silica.

**Figure 5:**
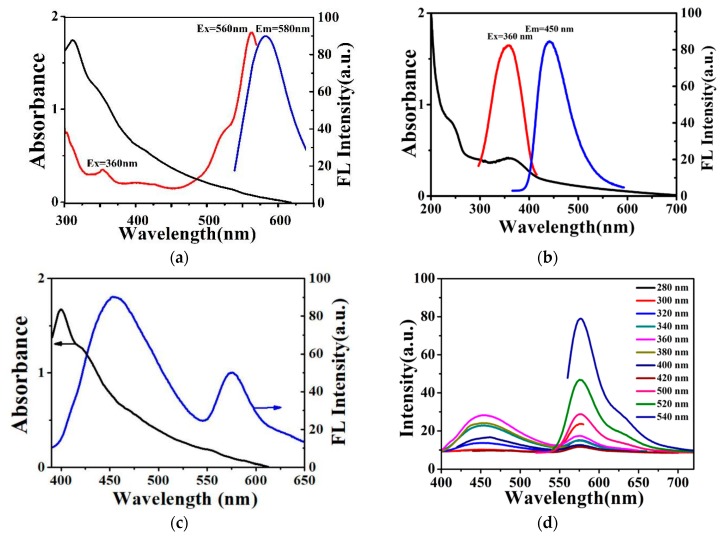
(**a**) Fluorescence spectra (blue line), excitation spectrum (red line), and absorption spectrum (black line) of RBS-doped silica microspheres; (**b**) fluorescence spectra (blue line), excitation spectrum (red line), and absorption spectrum (black line) of CA-AEAPMS; (**c**) fluorescence spectra (blue line) and absorption spectrum (black line) of dual-emission silica microspheres; (**d**) fluorescence spectra at different excitation wavelengths of dual-emission silica microspheres.

Figure 5a shows that the prepared RBS-doped silica microspheres have two excitation wavelengths, λ_ex_ = 360 nm and λ_ex_ = 560 nm, of which the optimal excitation wavelength λ_ex_ = 560 nm, and when excited using 360 nm and 560 nm excitation, the wavelengths are all λ_em_ = 580 nm [22]. And the absorption peak is around 360 nm. As shown in Figure 5b, the optimal excitation wavelength of CA-AEAPMS is 360 nm, which is consistent with the absorption peak. The emission wavelength at the 360 nm is 450 nm [21]. The fluorescence spectra and absorption spectrum of the dual-emission silica microspheres were shown in Figure 3c. A well-resolved dual emission spectrum was displayed at a single excitation wavelength (360 nm) (Figure 5c, blue line), where CA-AEAPMS emission peak is 450 nm and RBS emission peak is 580 nm, which demonstrated that the CA-AEAPMS was successfully coupled onto the RBS-doped silica microspheres. When dual-emitting fluorescent microspheres were excited with wavelength ranges of 280 nm to 540 nm, the wavelength dependence of the dual-emitting fluorescent microspheres in Figure 5d was obtained. The emission peaks at 450 nm and 580 nm were obtained with the change of excitation wavelength. Thus, we can achieve the control of color of the dual-emitting fluorescent microspheres through adjusting the intensity ratio of two emission peak.

### 3.2. Fluorescence Detection of Hg^2+^ and Biothiols Using the Dual-Emission Silica Microspheres

**Figure 6:**
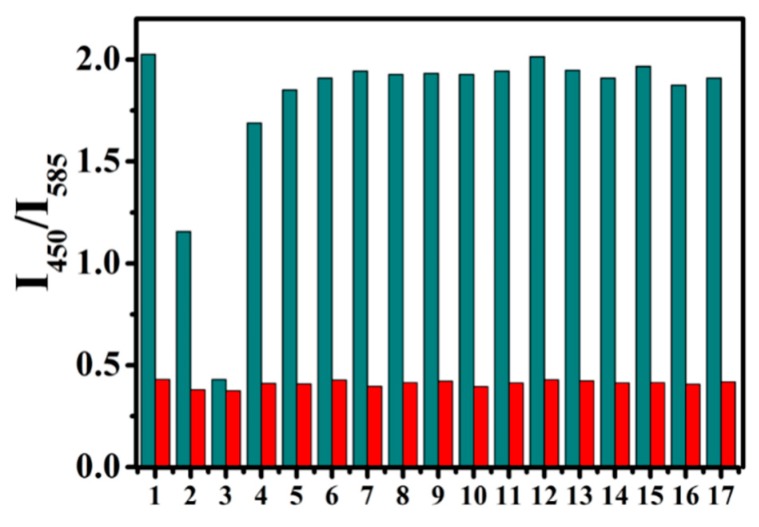
Fluorescence response of dual-emission microspheres (excited at 360 nm) to various metal ions and several common anions (20 μM, green bars) and the subsequent addition of Hg^2+^ (20 μM, red bars); 1 is a blank control, and 2 to 17 are Fe^3+^, Hg^2+^, Cu^2+^, Co^3+^, Al^3+^, Ca^2+^, Ni^2+^, Zn^2+^, Br^−^, I^−^, Na^+^, K^+^, CO_3_^2−^, Mg^2+^, Ba^2+^, and Ag^+^, respectively.

In order to evaluate the selectivity of dual-emission microspheres to Hg^2+^, the fluorescence intensity ratio (F_450_/F_585_) of dual-emission microspheres in the presence of other metal ions and several common anions was compared, such as Fe^3+^, Hg^2+^, Cu^2+^, Co^3+^, Al^3+^, Ca^2+^, Ni^2+^, Zn^2+^, Br^−^, I^−^, Na^+^, and K^+^ were studied under the same conditions. Figure 6 (green bars) shows the fluorescence of the dual-emission fluorescent microspheres is quenched by Hg^2+^ and Fe^3+^, and the quenching effect of mercury ions is particularly obvious. In the presence of other ions, the ratio of F_450_/F_585_ did not show a significant change compared to the original dual-emission microspheres, indicating good selectivity of the dual-emission microspheres for Hg^2+^. On this basis, the Hg^2+^ competitive experiment was carried out, as shown in Figure 6 (red bars), the ratios of F_450_/F_585_ decreased greatly, indicating that the presence of these ions have negligible interference with Hg^2+^ detection and further demonstrated the high specificity of the dual-emission microspheres for Hg^2+^. Because of the presence of large amount of amino groups on the surface of CA-AEAPMS, Hg^2+^ can selectively bind to CA-AEAPMS resulting in quenching of the blue fluorescence, and the red fluorescence of the RBS-doped silica core is still present, thereby achieving a ratio of fluorescence response to Hg^2+^.

**Figure 7:**
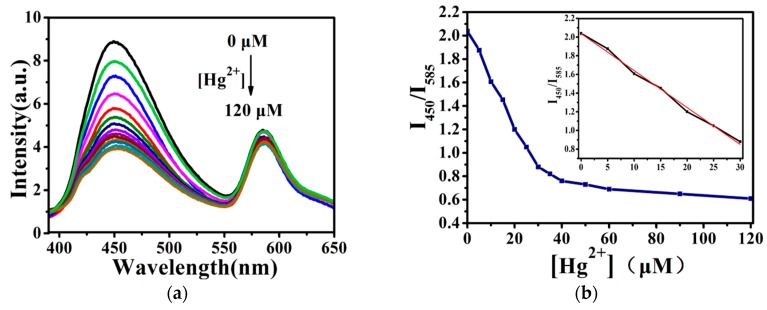
(**a**) Fluorescence spectra change of dual-emission silica microspheres upon addition of different concentrations of Hg^2+^ (excited at 360 nm). The concentration of Hg^2+^ from 0 to 120 μM; (**b**) fluorescence intensity ratio of F_450_/F_585_ versus Hg^2+^ concentration. Inset: linear fitting curve of F_450_/F_585_ versus a different concentration of Hg^2+^.

We investigated the response of the prepared dual-emission fluorescent microspheres toward different concentrations of Hg^2+^. The result was shown in Figure 7a. In the absence of Hg^2+^, two emission peaks at 450 and 580 nm were observed at 360 nm excitation due to the emission of CA-AEAPMS on the surface and the emission of RBS in the core of the silica microspheres, respectively. As the concentration of Hg^2+^ increases, the blue fluorescence at 450 nm decreased significantly in the range of 0–120 μM, indicating that the fluorescence of CA-AEAPMS was statically quenched by Hg^2+^, which is due to the complex formed between Hg^2+^ and amide group [18,19,20] of CA-AEAPMS. The red fluorescence at 580 nm was also found decreased as the concentration of Hg^2+^ increases, but the degree of reduction is much smaller than that of blue fluorescence.

Figure 7b shows the response of the fluorescence intensity ratio (F_450_/F_585_) versus Hg^2+^ concentration. The ratio of F_450_/F_585_ gradually decreased as the concentration of Hg^2+^ increases, we found that the fluorescence quenching in this system follows the Stern–Volmer equation:

I_0_/I = 1 + K_SV_ [Q] (1)

In the Stern–Volmer curve shown in Figure 5b, Hg^2+^ is not suitable for linear calibration over the entire concentration range of 0 to 120 μM. In contrast, Hg^2+^ can be fitted in the range of I_0_/I and Hg^2+^ concentrations ranging from 0 to 30 μM. A linear calibration plot of quantitative analysis with a linear regression equation of I_0_/I = −0.039 [Hg^2+^] + 2.031 (μM) (correlation coefficient R^2^ = 0.99394, *n* = 7). The higher concentration of Hg^2+^ deviates from the linearity based on 3δ/m (δ is the standard deviation of the double-emitting fluorescent microsphere signal (*n* = 7), m is the slope of the linear calibration chart), and the detection limit is 97 nM. Comparison of the present sensor with other existing Hg^2+^ sensitive probes based on silica was presented in Table 1 [23,24,25,26,27,28]. The results showed the detection limits of our present sensor were comparable and even better than those of other sensors, and the advantage of ratiometric detection is greater.

**Figure 8:**
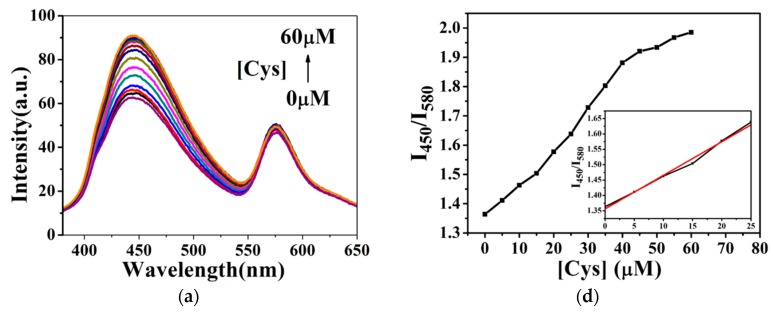

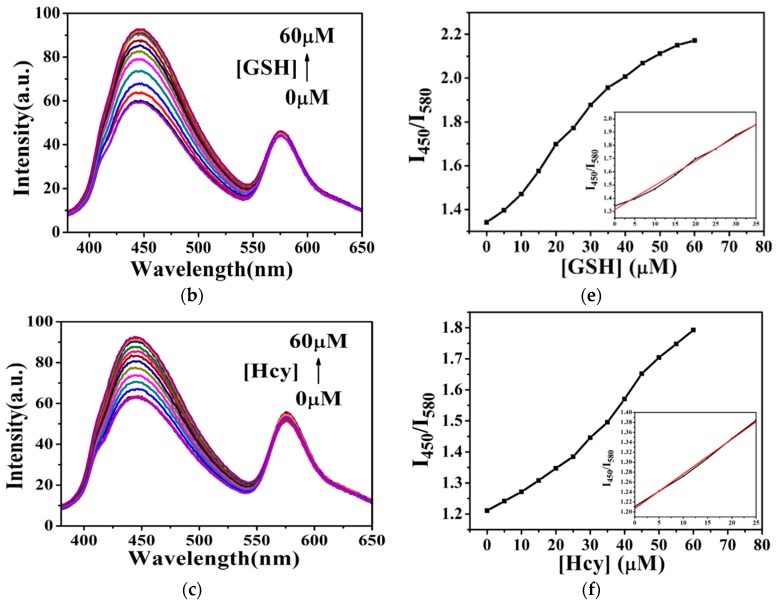
(**a**–**c**) Fluorescence spectra change of dual-emission silica microspheres upon adding different concentrations of Cys, GSH, and Hcy, after adding Hg^2+^ (excited at 360 nm). The concentration of Cys, GSH, and Hcy from 0 to 60 μM; (**d**–**f**) Fluorescence intensity ratio of F_450_/F_580_ versus the concentration of Cys, GSH, and Hcy. Inset: linear fitting curve of F_450_/F_580_ versus different concentration of Cys, GSH, and Hcy.

**Figure 9:**
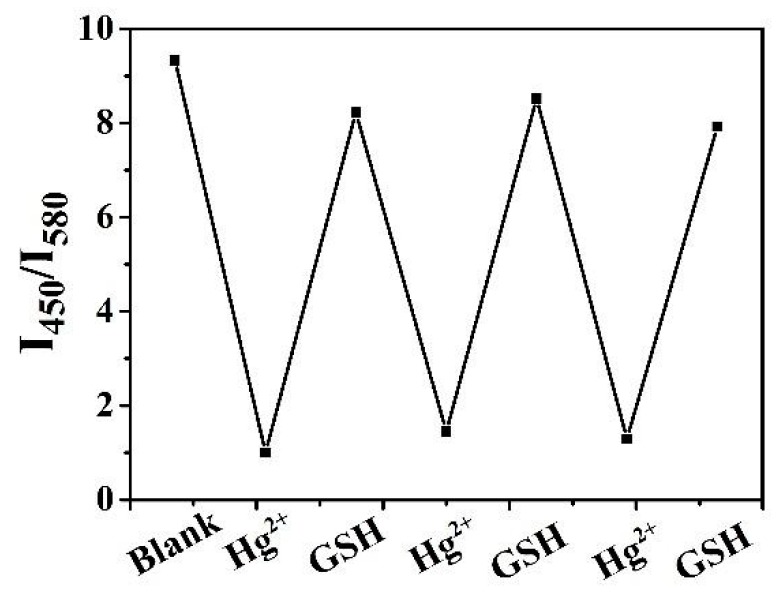
Reversibility of the dual-emission silica microspheres in deionized water by introducing equivalent mole of Hg^2+^ and GSH.

After the addition of Hg^2+^, the fluorescence was obviously quenched, then Cys, GSH, and Hcy were added to measure the fluorescence intensity of the solution, which are shown in Figure 8a–c. Hg^2+^ has high ability to bind to sulfhydryl groups in enzymes and proteins, nevertheless there is strong affinity between thiol groups and Hg^2+^. Therefore, three biothiols can separate Hg^2+^ from complexes with small molecules to restore fluorescence [5]. It can be seen that as the concentrations of Cys, GSH, and Hcy increase from 0 to 60 μM, the fluorescence intensity of the dual-emission microspheres gradually recovers, which obeys the Stern–Volmer equation. As shown in Figure 8d, the linear regression equation of Cys at 0–25 μM is I_0_/I = 0.011 [Cys] + 1.354 (μM) (correlation coefficient R^2^ = 0.99017, *n* = 6). From Figure 8e, the linear regression equation for GSH at 0–35 μM is I_0_/I = 0.017 [GSH] + 1.321 (μM) (correlation coefficient R^2^ = 0.99275, *n* = 8). In Figure 8f, the linear regression equation for Hcy at 0–25 μM is I_0_/I = 0.007 [Hcy] + 1.207 (μM) (correlation coefficient R^2^ = 0.99629, *n* = 6). At higher concentrations of Cys, Hcy, GSH, and linearity deviation, based on 3δ/m (δ is the standard deviation of the dual-emission fluorescent microsphere signal, m is the slope of the linear calibration chart), Cys, Hcy, GSH detection limits are 0.133 μM, 0.086 μ M, and 0.123 μM, respectively. In general, dual-emission fluorescent microspheres have high sensitivity to Cys, Hcy, and GSH detection. On the basis of this, the microspheres were tested for reusability, and GSH was selected as the removal agent for Hg. As shown in Figure 9, after adding Hg^2+^ (60 μM), the ratio of F_450_/F_585_ was reduced to the initial 78%, and then the same amount of GSH was added, the fluorescence intensity ratio was almost restored to the initial value. Then the solution was centrifuged and washed for cycle detection. The fluorescence of the dual-emission microspheres after three cycles shows excellent reversibility.

## 4. Conclusions

In summary, a ratiometric fluorescent nanosensor for the detection of Hg^2+^ and three biothiols has been designed and prepared by coupling CA-AEAPMS on RBS-doped silica microspheres. The obtained fluorescent microspheres exhibit characteristic emissions of RBS and CA-AEAPMS at excitation wavelength (360 nm), and can quench CA-AEAPMS emission when Hg^2+^ is combined, resulting in a ratio of fluorescence response of Hg^2+^ in aqueous solution. The selective experiments shows that this ratiometric fluorescence nanosensor is more selective for Hg^2+^ than other ions. The measurement of Hg^2+^ is sensitive and simple: the limit of detection down to 97 nM has been achieved and has excellent fluorescence reversibility. At the same time, the addition of three thiol-containing amino acids (Cys, Hcy, GSH) to the quenched fluorescence solution with Hg^2+^ can restore the fluorescence and detect them, and the detection limits of the three biothiols (Cys, Hcy, GSH) are 0.133 μM, 0.086 μM, and 0.123 μM, respectively.

## Figures and Tables

**Table 1:** Comparison among silica-based sensors for detection of Hg^2+^.

Materials	Detection Limit	Ref.
SiO_2_@mSiO_2_/Py-Si	1.1 × 10^−8^	[23]
QDs-MMS-Rh6G	2.5 × 10^−9^	[24]
SBA-15-HA	2.1 × 10^−7^	[25]
mesoporous silica nanoparticles	1 × 10^−8^	[26]
SiO_2_/AgNPs	5 × 10^−6^	[27]
SRhB-NH_2_	5 × 10^−7^	[28]
dual-emission silica microspheres	9.7 × 10^−8^	This work
